# Identification of tumor-specific T cell receptors from primary tumor-infiltrating lymphocytes

**DOI:** 10.1186/2051-1426-3-S2-P74

**Published:** 2015-11-04

**Authors:** Hiroyuki Kishi, Hiroshi Hamana, Kiyomi Shitaoka, Atsushi Muraguchi

**Affiliations:** 1 University of Toyama, Toyama, Japan

## 

Antigen-specific adaptive T cell therapy or T cell receptor (TCR) gene therapy is promising as a next generation tumor therapy. For the therapy, the production of tumor-specific T cells or their TCRs is prerequisite. Exploration of tumor-associated antigens, antigen-specific T cells or their TCR is usually restricted to the population with major HLA haplotypes, such as HLA-A24 for the Southeast Asian or HLA-A2 for Caucasian population. In this context, antigen-specific T cell therapy or TCR gene therapy is restrictive to those with major HLA haplotypes but is not beneficial for those with minor HLA haplotypes. In this study, we analyzed the TCRαβ repertoire of tumor-infiltrating lymphocytes (TIL) of B16F10 melanoma in C57BL/6 mice at single cell levels. We found the clonal expansion of T cells in TILs, but not in splenic T cells and detected tumor-specific T cells in those clonally expanded cells. Our single cell analysis system may greatly contribute to analyze tumor-specific T cells without restriction in HLA haplotypes, thus leading to personalized tumor immunotherapy in the future.

